# Catheter-related bloodstream infection caused by *Tsukamurella tyrosinosolvens* identified by *secA1*sequencing in an immunocompromised child: a case report

**DOI:** 10.1186/s12941-023-00651-6

**Published:** 2023-11-08

**Authors:** Shinsuke Mizuno, Yoshiyuki Tsukamura, Shuro Nishio, Toshiaki Ishida, Daiichiro Hasegawa, Yoshiyuki Kosaka, Tadasuke Ooka, Junichiro Nishi, Masashi Kasai

**Affiliations:** 1 Division of Infectious Disease, Department of Pediatrics, Hyogo Prefectural Kobe Children hospital, Kobe City, 6500047 Hyogo Japan; 2 Department of Inspection unit, Hyogo Prefectural Kobe Children hospital, Kobe City, Hyogo Japan; 3 Department of Hematology and Oncology, Hyogo Prefectural Kobe Children’s Hospital, Kobe City, Hyogo Japan; 4 Department of Microbiology, Kagoshima University Graduate School of Medical and Dental Sciences, Kagoshima, Japan

**Keywords:** *Tsukamurella tyrosinosolvens*, Actinomycetes, Catheter-related bloodstream Infection, Matrix-assisted laser desorption-ionization time-of-flight mass spectrometry, *secA1* gene sequencing, Pediatric

## Abstract

**Background:**

*Tsukamurella* spp. are obligate aerobic, gram-positive, non-motile, and slightly acid-fast bacilli belonging to the Actinomycetes family. They share many characteristics with *Nocardia*, *Rhodococcus*, *Gordonia*, and the rapidly growing *Mycobacterium* species. Therefore, standard testing may misidentify *Tsukamurella* spp. as another species. Accurate and rapid diagnosis is critical for proper infection management, but identification of this bacterium is difficult in the standard laboratory setting.

**Case presentation:**

A bloodstream infection caused by a gram-positive bacterium and related to a central venous catheter was identified in an immunocompromised 2-year-old girl. *Tsukamurella tyrosinosolvens* was identified by modified *secA1* sequencing. Antibiotic treatment and removal of the central venous catheter resolved the infection. Inappropriate management of the catheter during an overnight stay outside of the hospital was considered as a possible source of infection.

**Conclusions:**

*SecA1* sequencing may be a useful diagnostic tool in the identification of *T. tyrosinosolvens*. Providing proper central venous catheter care instructions to patients, their families, and medical staff is important for infection prevention.

## Introduction

Over the past several decades, advances in the treatment of malignant diseases have contributed to a significant reduction in overall cancer mortality [1]. However, these treatments are often associated with profound immunosuppression and an increased risk of infection, especially from opportunistic microorganisms. Early identification of these rare pathogens is critical for providing better treatment, but remains a challenge for microbiologists.

*Tsukamurella* spp. are obligate aerobic, gram-positive, non-motile, and slightly acid-fast bacilli belonging to the Actinomycetes family. They are found in a variety of environments, including soil, water, and arthropods [2, 3]. *Tsukamurella* spp. share many characteristics with *Nocardia*, *Rhodococcus*, *Gordonia*, and the rapidly growing *Mycobacterium* species. Therefore, standard tests may misidentify *Tsukamurella* spp. as one of these species [4, 5]. They were initially isolated as *Corynebacterium paurometabola* from bedbug mycetoma and ovaries in 1941 [6]. The name *Tsukamurella* comes from two microbiologists, Tsukamura and Mizuno, who described the first *Gordona aurantiaca* strain isolated from the sputum of a patient with a chronic lung disease in 1971 [7]. *Tsukamurella* currently has 16 species with validly published species, including *Tsukamurella tyrosinosolvens* (*T. tyrosinosolvens*), which was first described by Collins et al. in 1988 [8]. *Tsukamurella* spp. is a rare human pathogen associated with immunosuppressed patients, and a variety of infections have been reported, including pneumonia, brain abscesses, catheter-related bloodstream infections (CRBSIs), and ocular infections [3, 5–7, 9]. The gold standard for diagnosis and management remains to be determined. In addition, there is a lack of knowledge on infection prevention measures for this species.

Here, we present the case of an immunocompromised child with CRBSI caused by *T. tyrosinosolvens* identified by *secA1* sequencing. The infection was successfully treated with appropriate antibiotic therapy and source control. Inappropriate central venous catheter (CVC) management was considered as a possible source of infection.

## Case report

**Fig. 1:**
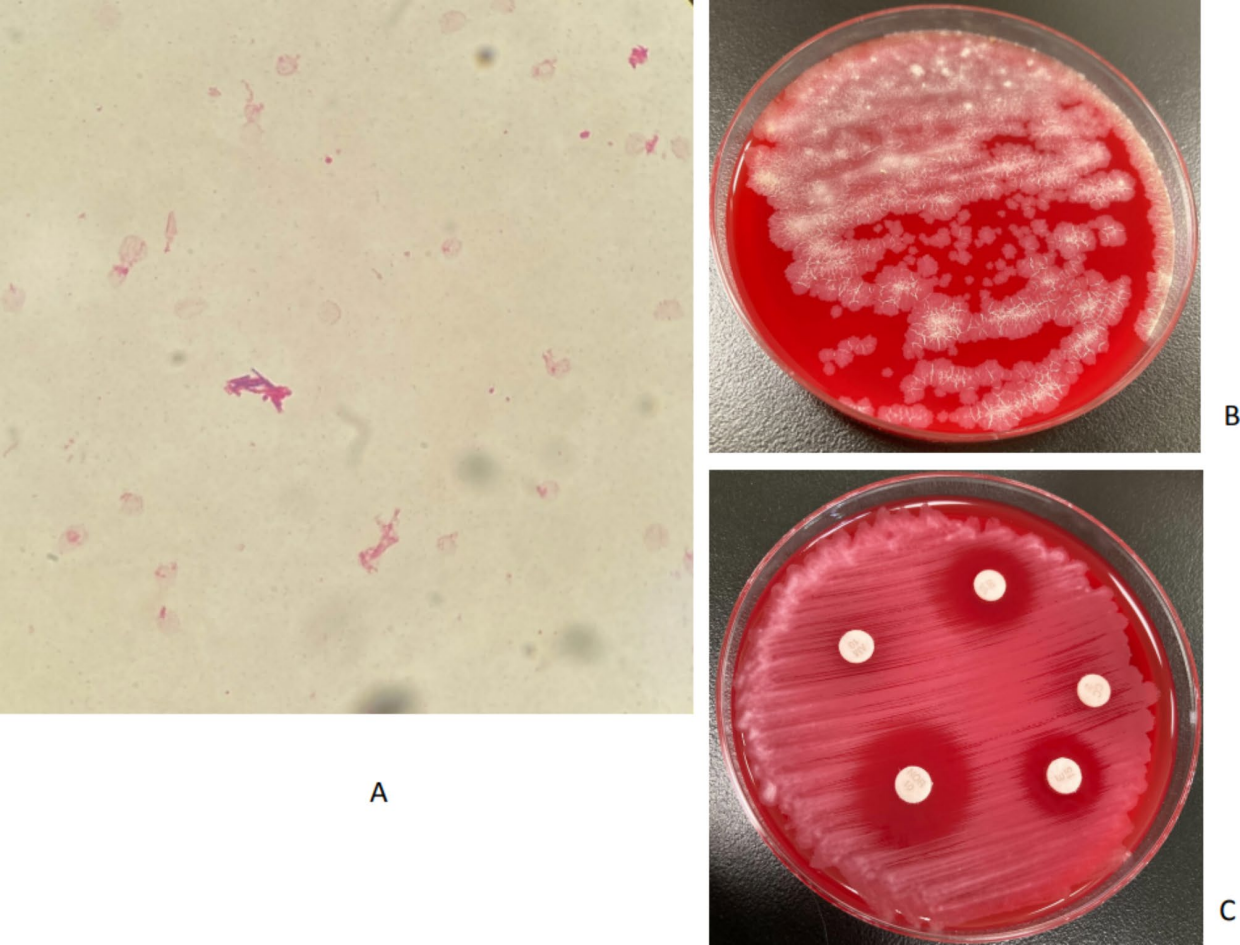
**A**: Gram staining of the isolates from aerobic blood cultures (× 1000 magnification). Numerous long, slightly curved, thin, non-branching, and gram-positive rods were confirmed. **B**: The development of flat, huge, rough, dry, and white- to light-cream-colored colonies was confirmed. **C**: The antibiotic disk diffusion susceptibility assay showed susceptibility to vancomycin, macrolides, and quinolones but resistance to amoxicillin and clindamycin. Diffusion results for isolates were based on the M24 Clinical and Laboratory Standards Institute (CLSI) document

A 2-year-old girl was diagnosed with acute B-cell precursor lymphoblastic leukemia (ALL). A CVC was inserted to initiate chemotherapy. The patient was treated with the Japanese Pediatric Leukemia/Lymphoma Study Group ALL-B19 protocols and was in complete remission after the induction phase. Near the end of the early intensification period, the patient developed a fever and general malaise (day 1). She temporarily stayed outside of the hospital for 3 days until the day before the fever developed. On physical examination, she was febrile with a temperature of 39.0 °C, tachycardic, and tachypneic. Cardiovascular examination was normal. Her lungs were clear on auscultation, and her abdomen was soft but not distended. No skin rashes were observed at the exit site of the catheter. However, the tape covering the catheter insertion site had peeled off when the patient returned to the hospital. Antibiotics were not administered, as the patient was non-neutropenic and showed no other signs or symptoms. On day 3, we obtained an aerobic blood culture from the CVC, which grew long, slightly curved, thin, non-branching and gram-variable rods on Gram staining (Fig. 1A). Kinyoun staining revealed weakly acid-fast positive rods. Vancomycin (VCM) and cefepime were administered intravenously. Two subsequent blood culture sets from the CVC taken on day 2 and three blood culture sets taken from both the peripheral vein and CVC on day 3 were again positive for gram-positive rods.

Blood samples cultured after 48 h of incubation at 35 °C in 5% CO_2_ on chocolate and blood agar showed flat, huge, rough, dry, and light-cream-colored colonies (Fig. 1B). The isolate was susceptible to VCM, macrolides, and quinolones but resistant to amoxicillin and clindamycin, as determined by the disk diffusion test based on the M24 Clinical and Laboratory Standards Institute (CLSI) document (Fig. 1C). Cefepime was discontinued, and the CVC was removed on day 3. Culture of the removed catheter also showed the growth of the same colonies, suggesting a CRBSI. A blood culture performed on day 4 was negative, and the patient’s condition improved thereafter. VCM was continued for 14 days after the confirmation of negative blood culture. No clinical relapse of bloodstream infection was observed over the following 3 months.

The isolated strain was assessed using the Bruker Biotyper matrix-assisted laser desorption-ionization time-of-flight mass spectrometry (MALDI-TOF-MS) system (Bruker Daltonics). MALDI-TOF-MS revealed *Tsukamurella tyrosinosolvens* with the scores obtained ranging from 2.00 to 2.299, allowing for a highly probable genus and probable species identification level (Table 1). However, other species also showed similarly high scores. The isolates from the blood culture were referred to the microbiology laboratory and additional molecular tests were performed to confirm the species. *T. tyrosinosolvens* was identified using polymerase chain reaction-restriction fragment length polymorphism analysis of *secA1*. A region of approximately 900 base pairs was extracted from *secA1* alignment of each *Tsukamurella* species and redesigned into two candidate primers: tsuka_secA1_F1 (5’-GCGACAAGGACTACATCGTC-3’) and tsuka_secA1_R1 (5’-ACGAACTTGTTGTCGATCGG-3’). Sequences with these primers and those obtained from our isolate were compared with GenBank entries (GenBank accession no. U90204), using the BLASTN analysis in combination with previously reported criteria, and showed over 99.5% homology with *T. tyrosinosolvens* and less than 96% homology with other *Tsukamurella* species.

**Table 1:** Matrix-assisted laser desorption-ionization time-of-flight mass spectrometry (MALDI TOF-MS) biotyper system genus-level identification.

Matched Pattern	Score Value	NCBI Identifier
*Tsukamurella tyrosinosolvens* DSM 105033 DSM	2.17	57704
*Tsukamurella paurometabola* DSM 46042 DSM 2	1.95	2061
*Tsukamurella tyrosinosolvens* DSM 45557 DSM	1.79	57704
*Tsukamurella tyrosinosolvens* DSM 44370 DSM	1.65	57704
*Tsukamurella paurometabola* DSM 46042 DSM	1.65	2061
*Tsukamurella tyrosinosolvens* DSM 44330 DSM	1.63	57704
*Tsukamurella tyrosinosolvens* DSM 44316 DSM	1.62	57704
*Tsukamurella pulmonis* CCUG 47079 CCUG	1.55	47312
*Tsukamurella pulmonis* DSM 44142T DSM	1.51	47312
*Tsukamurella inchonensis* DSM 43246 DSM	1.47	42777

Abbreviations: NCBI, National Center for Biotechnology Information.

## Discussion

In the current case, CRBSI caused by *T. tyrosinosolvens*, which is very rare in children, was identified using a modified *secA1* sequence. The patient was successfully treated with VCM monotherapy and CVC removal. Improper CVC management during the overnight stay outside of the hospital may have caused the infection.

*T. tyrosinosolvens* and related species are sometimes misdiagnosed for contaminant microorganisms or other species because they are difficult to identify with standard laboratory tests. Delays in accurate diagnosis and misdiagnoses can lead to suboptimal antibiotic selection, delayed treatment, which in turn may lead to systemic infection and poor prognosis [3, 5, 9–12]. A method for identifying uncommon pathogens is MALDI TOF-MS, which provides reasonably rapid genus-level identification, and, therefore, better patient care [13]. However, MALDI TOF-MS is not able to identify *Tsukamurella* species as there are few genetic differences between them [5, 10–12]. Species-level identification is important as it can contribute to the correct epidemiological characterization of unusual pathogens. Several tests have been successful for species identification, including 16 S rRNA gene sequencing and sequencing of several target genes [14, 15]. However, previous studies have shown that the majority of *Tsukamurella* species have highly similar 16 S rRNA gene sequences, and as a result, it has been found to be insufficiently discriminative in identifying *Tsukamurella* species [3, 5, 9, 11, 14]. By contrast, the *secA1* sequence has been shown to be suitable for the discrimination of clinically important *Tsukamurella* spp [16]. In this case, modified *secA1* sequencing was necessary for species identification.

Most reported cases of *T. tyrosinosolvens* infection have been related to bacteremia due to intravascular prosthetic devices, immunosuppression following hematological malignancy or post chemotherapy, and graft-versus-host disease after bone marrow transplant [3, 17, 18]. The optimal management of *Tsukamurella* infections has yet to be determined. To date, the CLSI document M24 provides criteria for susceptibility testing of *Tsukamurella* species by the broth microdilution method [19]. If there is doubt about the results of the broth microdilution method, CLSI recommends the disk diffusion test to be performed. In most case reports, susceptibility to amikacin, clarithromycin, imipenem, ciprofloxacin, and trimethoprim-sulfamethoxazole and resistance to penicillin, cefoxitin, and expanded-spectrum cephalosporins have been reported in *Tsukamurella* isolates [3, 9]. Based on the treatment principles for nocardiosis and atypical mycobacterial infections, a number of antimicrobial agent combinations have been proposed as potential treatments for *Tsukamurella* infections [3, 6]. Our isolate was susceptible to VCM and quinolones, in line with the results of previous reports. The patient in this case wastreated with VCM monotherapy for 14 days and CVC removal with good outcome.

A previous study identified multiple inappropriate infection control practices, with the most likely cause being improper management of CVC lines [20]. In the current case, the patient had stayed outside of the hospital for 3 days prior to onset and we suspect that the infection was caused by improper CVC management during this time.

In conclusion, in addition to rapid genus identification by MALDI TOF-MS, *secA1* sequencing may be a useful diagnostic tool in the confirmation of *Tsukamurella* spp. Rapid identification facilitates faster treatment, and may lead to a good prognosis. Providing proper management of CVCs is essential for prevention of infection.

## Data Availability

The datasets generated and/or analyzed during this study are available from the corresponding author upon reasonable request.
